# Dermal cylindroma of the external auditory canal

**DOI:** 10.4322/acr.2023.447

**Published:** 2023-10-23

**Authors:** Vivek Dokania, Indranil Mukherjee

**Affiliations:** 1 Asian Super Specialty Hospital, Department of Otolaryngology-Head & Neck Surgery, Dhanbad, Jharkhand, India; 2 Gouri Devi Institute of Medical Science and Hospital, Department of Otolaryngology-Head & Neck Surgery, Durgapur, India

**Keywords:** Ear, Ear canal, Ear neoplasms

## Abstract

External auditory canal (EAC) cylindroma is a rare tumor that mainly presents as a painless mass over the lateral aspect of the ear canal. They have been designated under different nomenclatures in the literature, and controversies persist about their etiology and histogenesis. Moreover, a clinical diagnosis of EAC cylindroma is often challenging because of their rarity and a close resemblance with other adnexal benign and malignant tumors. None of the previous authors have extensively reviewed the dermal cylindroma of the EAC. We provide an extensive review involving PubMed and Google Scholar and report by Preferred Reporting Items for Systematic Reviews and Meta-Analyses standards. A total of 8 cases are included in the current study. The mean age is 55.13 years. There are six females and two males. The left and right ear are involved in 62.50% and 37.50% of cases, respectively. The most common sign/symptom is painless mass (50%). Five authors reported a primary lesion (62.50%), while the remaining 3 reported a recurrent tumor (37.50%). Benign versus malignant cylindroma is reported in 87.50% and 12.50% of cases, respectively. All, except one case, reported a solitary swelling. Surgical excision was employed in all the cases. Primary defect closure versus defect closure with local/distant skin graft /flap is utilized in 37.50% and 62.50% of cases, respectively.

## INTRODUCTION

Cylindromas are rare adnexal skin tumors mostly found in the head and neck region. They are mostly seen as solitary lesions, although multiple lesions over the scalp have been associated with Brooke-Spiegler syndrome.

Only 6 percent of all cylindromas affect the ear.^1^ However, the reported incidence might not be precise, as various glandular tumors, including adenoid cystic carcinoma, have also been erroneously termed cylindroma. These tumors arise from the cartilaginous portion of the EAC. The lateral or cartilaginous EAC contains hair follicles and is associated with ceruminous glands from which these tumors arise. Controversy persists about the terminology, etiology, and histogenesis of this tumor. This prompted us to review articles on the dermal cylindroma of the EAC.

## METHODOLOGY

**Figure 1:**
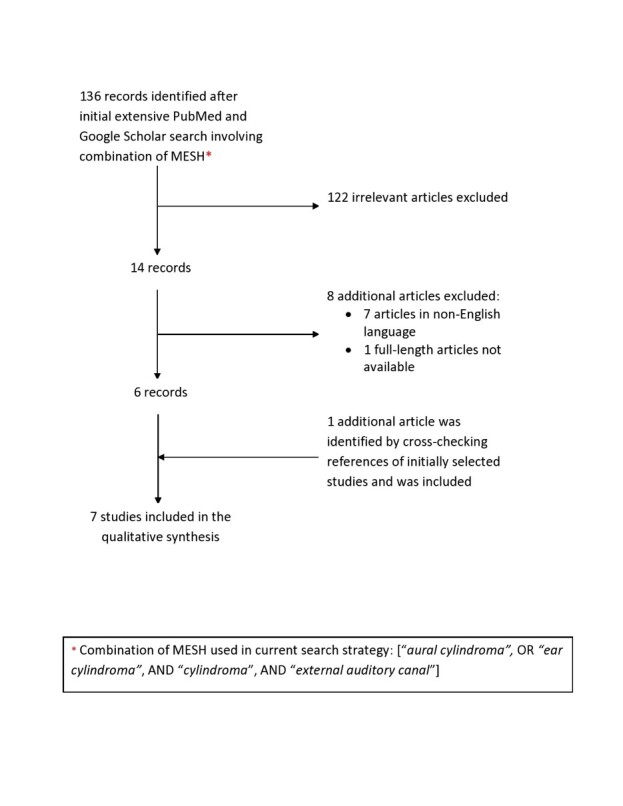
Literature search flow diagram based on the Preferred Reporting Items for Systematic Reviews and Meta-Analyses (PRISMA).

An extensive search of two medical databases (PubMed and Google Scholar) was performed by the Preferred Reporting Items for Systematic Reviews and Meta-Analyses (PRISMA) (Figure 1) standards in December 2022. The databases were searched for full-length articles and abstracts published in the English language and confined to human subjects, using the following Medical Subject Headings (MeSH): “*aural cylindroma*”, OR “*ear cylindroma*”, AND “*cylindroma*”, AND “*external auditory canal*”. Tumors in the “external auditory canal with or without pinna extension” were included. Both benign and malignant tumors were included. No age, race or demographic filters were applied. Only the articles published in English literature and confined to humans were included. Information from the included articles was collected in a pre-designed Microsoft Excel (Microsoft Corporation, USA) spreadsheet. No ethical approval was obtained as it was not required for this study.

## CASE REPORT

**Figure 2:**
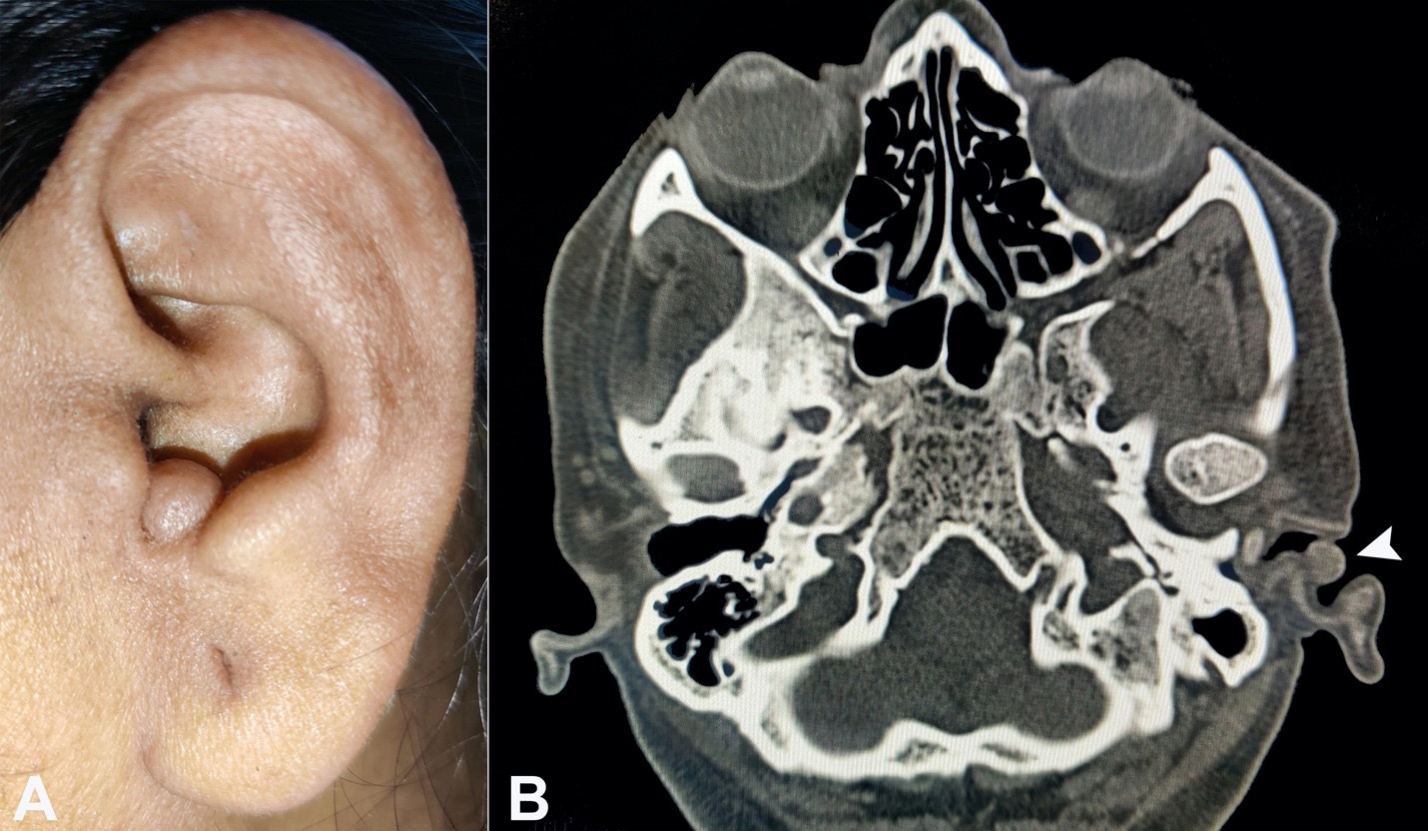
**A -** gross view of the lesion over the left ear’s cavum concha; **B -** head computed tomography, axial view showing a homogeneous, well-circumscribed, round lesion over the lateral aspect of the left external auditory canal and adjacent to cavum concha (arrowhead).

A 48-year-old female presented with a painless nodule over the cavum concha of the left ear for one year. The lesion was 10 x 8 mm, firm, non-tender, and gradually progressed to the present size (Figure 2A). She had no other aural complaints. She was otherwise healthy and did not report a history of alcohol consumption or smoking. No genetic or syndromic abnormalities were reported from her family.

The computed tomography showed a homogeneous, well-circumscribed, round lesion over the lateral aspect of the left external auditory canal adjacent to the cavum concha. The lesion did not seem to infiltrate the underlying cartilage or erode the overlying skin (Figure 2B).

**Figure 3:**
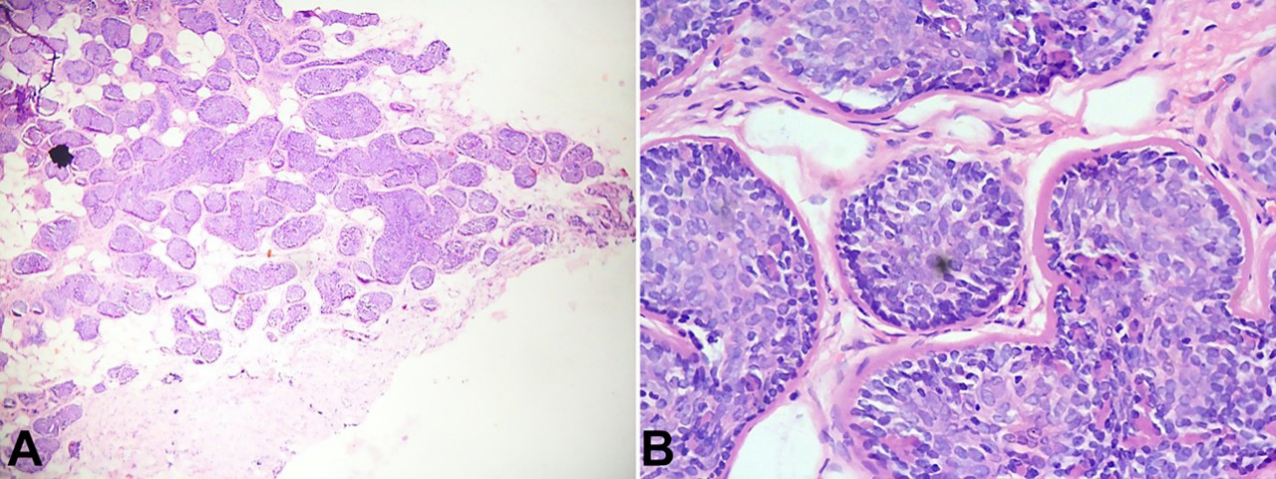
Photomicrographs of the tumor. **A -** compact nests of basaloid cells arranged together resembling a jigsaw puzzle pattern (H&E; 100X); **B -** nests are composed of an outer layer of peripheral palisading cells with small, dark nuclei and centrally located ductal cells with larger, pale nuclei. These nests are surrounded by a thickened, hyalinized membrane (H&E; 400X).

The lesion was excised entirely after raising the skin flap. The histopathological examination of the resected tissue revealed a tumor composed of compact nests of basaloid cells with cylindrical outlines and surrounded by a thickened, hyalinized membrane. The nests resemble a jigsaw puzzle and were composed of an outer layer of peripheral palisading cells with small, dark nuclei and centrally located ductal cells with larger, pale nuclei. Focal luminal differentiation was seen (Figure 3).

**Figure 4:**
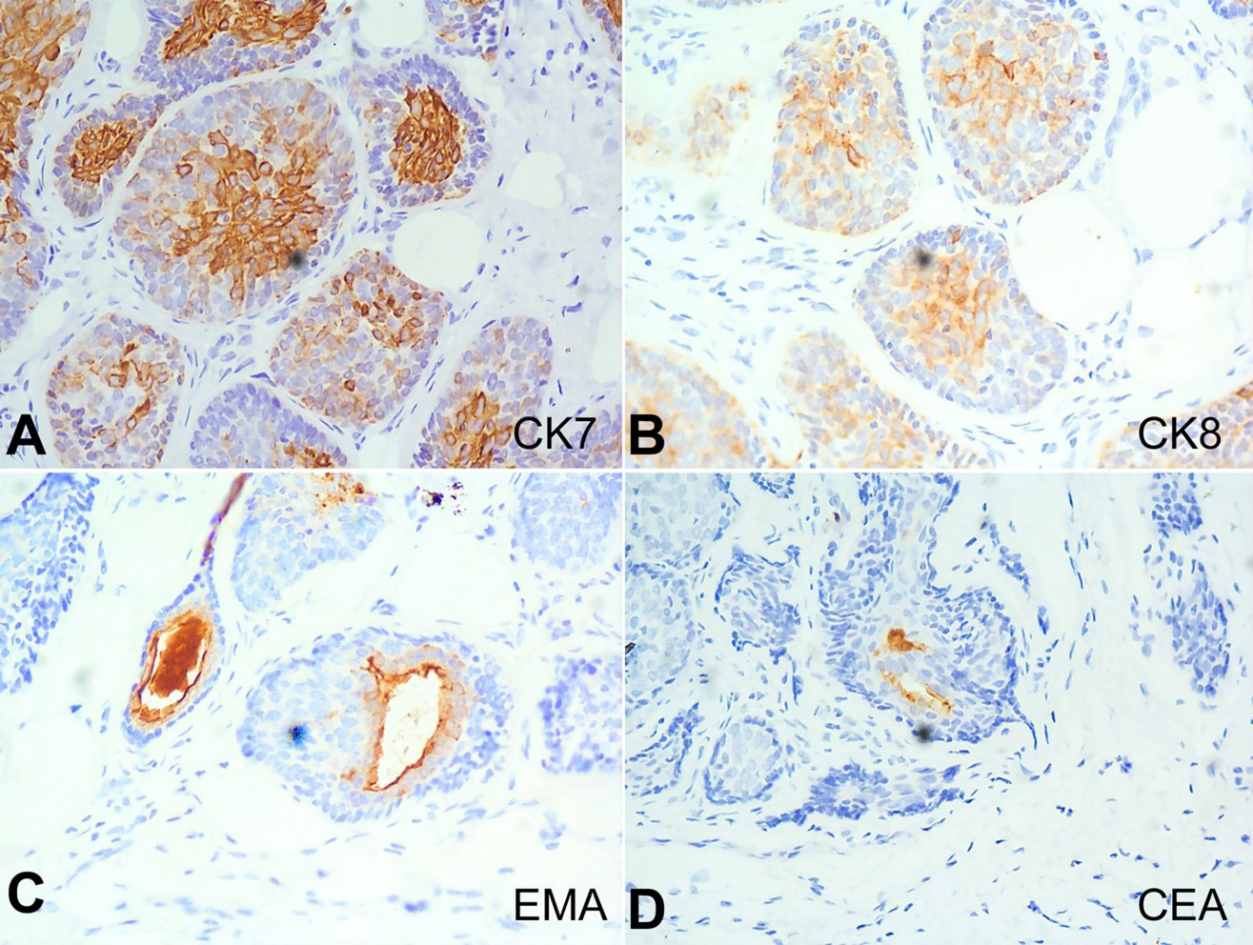
Photomicrographs of the tumor. Immunohistochemistry. **A -** central cells are immuno-positive for CK7; **B -** central cells are immuno-reactive for CK8 /18; **C -** luminal areas showing EMA immuno-reactivity; **D -** luminal areas showing CEA immuno-positivity.

The central basaloid cells were immuno-reactive for CK7 and CK8/18, with luminal differentiation areas exhibiting immunopositivity for CEA and EMA (Figure 4). The tumor cells were negative for CK20, BerEP4, CD10, and androgen receptors. There was no evidence of malignancy. The final histopathological report diagnosed the mass as “benign dermal cylindroma of the left external auditory canal.”

## RESULT

8 cases were included in the current review (seven published cases and one illustrative case). Demographic, clinical, and treatment findings were documented (Table 1).^2-8^

**Table 1:** Summary of cases of “external auditory canal dermal cylindroma”

Ref.	Age (years)	Sex	Side	Clinical symptoms	P/R lesion	Treatment
2	35	M	Left	Ear pain and discharge. Decreased hearing	R	Wide local excision with canal cartilage & defect repair with full-thickness forearm skin graft
3	53	F	Right	Painless mass	R	Wide local excision & defect repair with post-aural skin graft
4	44	F	Right	Painless mass	P	Wide local excision with tragal & surrounding cartilage and defect repair with full-thickness skin graft
5	61	F	Left	Painless mass	P	Complete excision with removal of tragal and conchal cartilage, and repair with local rotational skin flap
6	58	F	Left	Aural fullness, Tinnitus, Otalgia	P	Primary local excision followed by secondary full-thickness removal followed by Moh’s technique & defect repair with split-thickness skin graft
7	75	M	Right	Aural fullness	P	Complete excision
8	67	F	Left	Decreased hearing	P	Complete excision
IC	48	F	Left	Painless mass	P	Complete excision

IC = index case; F = female; M = male; P/R = primary/recurrent; P = primary; R = recurrent; Ref = reference.

There were six females and two males (female/male ratio: 3/1). The ages ranged from 35 to 75 years (Mean: 55.13 years, SD: 12.89). The cases were reported from five different countries: USA (n = 3), India (n = 2), UK (n = 1), Germany (n = 1), and Malaysia (n = 1).

The lesion involved the left ear in five (62.50%) cases and the right ear in three (37.50%) cases. Five cases presented a primary lesion (62.50%), while the remaining 3 cases presented a recurrent lesion (37.50%).

Clinical symptoms/signs were present in the following frequencies: Painless mass (50%), ear pain/otalgia (25%), decreased hearing (25%), aural fullness (25%), tinnitus (12.5%), and ear discharge (12.5%).

Except for Ramadass,^2^ all other cases presented a solitary aural lesion. Ramadass^2^ reported multiple firm masses at 3, 5, and 9 o’clock positions in the EAC. Similarly, all articles except one presented with a benign tumor variant. Mashkevich et al.^6^ reported a case of malignant cylindroma of the EAC.

Surgical excision was employed in all the cases. In three cases, wide local excision with additional removal of surrounding EAC/pinna cartilage was utilized.^2,4,5^ Primary defect closure was done in 3 lesions, while defect closure with local/distant skin graft or flap was utilized in the remaining five cases.

## DISCUSSION

The first case of dermal cylindroma was reported in 1842.^1^ Although the first case of falsely designated EAC cylindroma for an adenoid cystic carcinoma (ACC) was described in 1963.^9^ Since then, various glandular lesions in the EAC have been wrongly designated as cylindroma and different terminologies have been interchangeably used in the literature. To the best of our knowledge, the first true case of EAC dermal cylindroma was reported by Ramadass^2^ in 1966.

Dermal cylindromas are mostly solitary, slow-growing, and asymptomatic lesions. 87.50% of lesions in our study were solitary tumors. Multiple lesions are mostly inherited and associated with familial cylindromatosis or Brooke-Spiegler syndrome.^10^ EAC cylindromas are rare tumors and are mostly found on the lateral cartilaginous portion of the canal.

The etiopathogenesis of this tumor is unclear; both eccrine and apocrine origins have been discussed in the literature. Although, some authors have postulated that the tumor originates from a common progenitor prior to differentiation.^8^

Dermal cylindromas are mostly benign lesions. Nonetheless, malignant degeneration into adenoid cystic carcinoma and malignant cylindroma is possible. Clinical signs, such as bleeding, ulceration, or sudden growth, may suggest a malignant transformation.^7^ Histological features such as infiltrative pattern, loss of jigsaw pattern, or infiltration of hyaline sheath and peripheral structures suggest malignant transformation.^11^

Differentials include other benign appendage tumors, such as trichoepitheliomas, syringomas, ceruminous adenomas, and epidermal cysts. Besides, neurofibromas, adenocarcinoma, ACC, pleomorphic adenoma, and basal cell epitheliomas may even clinically resemble cylindromas.^4,12^

Despite its benign nature, cylindroma has a recurrence rate of over 40%, and it can also undergo malignant transformation. Therefore, a complete surgical excision with tumor-free margins and a close follow-up is important.^7,8^ Besides surgery, treatment with CO_2_ laser and radiation therapy with 4000 to 6000 rads has also been reported for multiple or massive cylindromas.^4,7^

## CONCLUSION

EAC cylindroma should be considered amongst the differential for any solitary, painless tumor at the lateral aspect of the external ear canal. They pose a diagnostic challenge because of their close clinical resemblance with other benign and malignant EAC lesions. An early diagnosis and optimal surgical intervention are important to prevent high recurrence associated with the tumor. More studies are required to shed light on the persisting controversy related to the etiology and histogenesis of this rare tumor.
